# Mayoral Health Meeting

**Published:** 1920-05-08

**Authors:** 


### Mayoral Health Meeting.

The Lord Mayor will preside at a meeting to be held at the Mansion House on May 11, at 3 p.m., for the purpose of bringing before the Lord Mayors and Mayors of the country the objects and scope of the People's League of Health. This body was founded by Miss Olga Nethersole, with a view to promoting sound education of the people in matters pertaining to health. In such a cause the interest and sympathy of the municipal and other local authorities are necessary, no less than the co-operation of the medical profession. At the Lord Mayor's meeting on May 11 distinguished medical members of the Council of the People's League of Health will emphasise the need for education in health, will describe the methods of action proposed by the League, and will invite the interest and assistance of the Lord Mayors and Mayors wherever the latter think that the League can be of service to them.

